# Validation of the Clinical Global Impression—Corrections Scale (CGI-C) by Equipercentile Linking to the BPRS-E

**DOI:** 10.3389/fpsyt.2020.00180

**Published:** 2020-03-20

**Authors:** Roland M. Jones, Cory Gerritsen, Margaret Maheandiran, Alexander I. F. Simpson

**Affiliations:** Centre for Addiction and Mental Health (CAMH) and University of Toronto, Toronto, ON, Canada

**Keywords:** CGI-C, BPRS-E, correctional psychiatry, prison, mental health, assessment, rating scale, severity

## Abstract

**Background:** The Clinical Global Impression—Corrections (CGI-C) is an adaptation of the severity scale of the Clinical Global Impression for use in correctional facilities. Although it has been shown to have good inter-rater reliability, there have been no validation studies of this instrument.

**Method:** We analyzed data from 726 initial assessments of persons detained in two correctional facilities and compared clinician's ratings for the CGI-C and modified Brief Psychiatric Rating Scale-Expanded (BPRS-E). We used equipercentile linkage and Spearman correlations to investigate concordance in the total sample, by diagnostic groups, and by gender.

**Results:** We found that the CGI-C scores and BPRS-E scores among persons in remand settings were significantly correlated (ρ = 0.51, *p* < 0.001) and that correlations were the same for men and women. We found that points of equivalence can be reliably found between the two scales using equipercentile linkage, and that those with psychotic disorders had lower BPRS-E scores than those with mood/anxiety/situational stress for equivalent CGI-C scores.

**Conclusion:** Overall, CGI-C ratings correspond well to BPRS-E ratings for both men and women remand prisoners across diagnoses, and the CGI-C appears to be a valid tool for the assessment of severity of symptoms in this setting.

## Background

There is a high prevalence of mental illness among prisoners world-wide (1), and there are significant barriers to the provision of effective care, including challenges in identifying and assessing those who are in need of treatment (2). Several scales have been designed to screen for mental disorder in correctional institutions (3) but there has been little research into the measurement of severity of mental disorder in correctional facilities. Existing tools that are used in general settings for measuring symptom severity such as the Positive and Negative Symptom Scale (PANSS) (4), or the Hamilton Depression Rating Scale (5) could also be used in correctional settings. However, given the operational challenges of providing mental health care in correctional settings, and the high severity of illness and behavioral disturbance due to mental illness among many prisoners, there is a need for a brief assessment tool that can be used in routine practice to assess severity and to monitor change (6, 7).

The Clinical Global Impression—Corrections (CGI-C) (8) was developed as an adaptation of the severity scale of the original Clinical Global Impression (9) (CGI-S) as a brief, reliable tool to measure overall symptom severity of individuals in correctional settings, and to be used by clinicians of different disciplines. It is, as far as we are aware, the only symptom rating scale that has been developed for use in correctional settings. Global symptom severity is rated on a 7-point scale based on direct observation and information from correctional officers or other sources. The tool has been shown to have good inter-rater reliability (10), and has also been translated into German (11) with similar inter-rater reliability.

Comparisons between the original CGI-S and the Brief Psychiatric Rating Scale (BPRS) (12, 13) and the Positive and Negative Symptom Scale (PANSS) (4, 12, 14, 15) have shown a good overall level of concordance among people with schizophrenia. Variations in the degree of concordance have however been found for different symptom clusters, with a greater degree of concordance with positive symptoms, rather than negative or depressive symptoms (14, 16).

Although adapted from the original CGI-S, the CGI-C has not previously been validated by direct comparison with any other rating scales, nor have there been any validation studies of symptom severity rating scales within correctional facilities. Our aim was to validate the CGI-C by comparing CGI-C ratings with BPRS-E ratings among a consecutive sample of remand prisoners, and to investigate whether there were differences in concordance on ratings of primarily affective of psychotic disorders.

## Methods

We analyzed data collected during routine clinical care within two provincial correctional facilities in Southern Ontario, Canada; Vanier Centre for Women (VCW), and the Toronto South Detention Centre (TSDC). In these facilities, all inmates are screened at reception by correctional health staff using the Brief Jail Mental Health Screen (17). Those screening positive are then referred to the Forensic Early Intervention Service (FEIS) for further assessment, and are typically assessed within 3 days of arrival at the facility. FEIS clinicians (comprising nurses, social workers, and occupational therapists), carry out assessments using the Jail Screening and Assessment Tool (JSAT) (18) and CGI-C, having received training in using these tools. The JSAT contains a modified BPRS scale (BPRS-E) which comprises 24 items, which are rated 0 (not present), 1 (partially present) or 2 (present). The JSAT also requires clinicians to categorize the putative mental disorder into non-mutually exclusive symptom groups: Situational Stress/Depression; Possible Anxiety/Mood Disorder; History Psychosis/Bipolar Disorder; Possible Recurrent Psychotic Symptoms; Active Current Psychosis; Intellectual Disability/Brain Damage; and Personality Disorder Traits.

We first carried out analyses on the entire sample, irrespective of symptom group. We then created categories of “Mood/Anxiety/Situational Stress” by combining Situational stress/Depression and Possible Anxiety/Mood Disorder, and “Psychotic illness” by combining History Psychosis/Bipolar Disorder, Possible Recurrent Psychotic Symptoms and Active Current Psychosis. There were insufficient participants with a recorded singular diagnostic category of either intellectual disability / brain damage or personality disorder traits to analyse separately. For the purposes of our secondary analysis by non-overlapping diagnostic groups, we excluded participants who were recorded as having both “Mood/Anxiety/Situational Stress” and “Psychotic illness” leaving diagnostic groups of either “Mood/Anxiety/Situational Stress” (*n* = 290) or “Psychotic illness” (*n* = 185). We extracted data from clinical records for all inmates in which a BPRS-E and CGI-C were recorded on the same day between September 1, 2017 and June 29, 2019.

### Statistical Analyses

Equipercentile linking is the preferred method of comparing one scale against another (19–21), and is a technique that establishes points of equivalence between scores on both measures that have the same percentile rank (22). We carried out analyses using the Equate package in R (23) based on the test equating theory described by Kolen and Brennan (22). Single group design was utilized as both scores were measured on the same sample. We first carried out equipercentile linking on the total sample, and then separately for those who were categorized as having a psychotic illness or a mood/anxiety/situational stress disorder.

We also investigated correlations between the CGI-C and BPRS-E. Spearman's rho was used given the ordinal nature of CGI-C data and positive skew observed in both measures. Bivariate outliers were defined as observations with a Mahalanobis distance ≥13.82 (corresponding with *p* ≤ 0.001). No such observations existed and all leverage values were ≤0.02, so all observations were included in the analyses. Separate correlations were then calculated by diagnosis as defined above. These analyses were performed using the Statistical Package for Social Sciences, Version 25 (24).

## Results

The sample consisted of 429 (59.1%) men and 292 (40.2%) women. Five additional inmates identified as having a gender other than male or female were also included in the total sample (*n* = 726). The mean age of the sample was 35.4 years (SD = 10.6) (see Table 1). Most patients received a diagnosis of a mood related illness (64.7%), and 50.2% of patients were diagnosed with a psychotic illness. Other diagnoses included personality disorders (19.9%) and intellectual disability/brain damage (2.6%). For all patients, the mean CGI-C score was 3.2 (SD = 1.3) and the median score was 3 (IQR = 2). The mean total modified BPRS-E score for all patients was 8.8 (SD = 7.6), and the median score was 6 (IQR = 8.75).

**Table 1:** Participant characteristics.

	**Whole sample (*n* = 726)**	**Males (*n* = 429)**	**Females (*n* = 292)**
Age: mean (SD)	35.4 (10.6)	36.0 (11.2)	34.5 (9.7)
BRSE-E Total: mean (SD)	8.8 (7.6)	8.3 (7.4)	9.6 (7.9)
CGI-C: mean (SD)	3.2 (1.3)	3.4 (1.4)	3.0 (1.1)
**Putative diagnoses**			
Situational stress/depression	404 (55.6%)	201 (46.9%)	199 (68.2%)
Possible anxiety/mood disorder	269 (37.1%)	134 (31.2%)	133 (45.5%)
History psychotic/bipolar disorder	123 (16.9%)	74 (17.2%)	48 (16.4%)
Possible recurrent psychotic symptoms	233 (32.1%)	137 (31.9%)	95 (32.5%)
Active current psychosis	69 (9.5%)	49 (11.4%)	20 (6.8%)
Intellectual disability/brain damage	19 (2.6%)	10 (2.3%)	9 (3.1%)
Personality disorder traits	101 (13.9%)	62 (14.5%)	36 (12.3%)

CGI-C scores correlated significantly in the sample for modified BPRS-E total scores (ρ = 0.51, *p* < 0.001). There was no difference in correlations between males (ρ = 0.54, *p* < 0.001), and females (ρ = 0.54, *p* < 0.001). We also found significant correlations between CGI-C and BPRS-E among those with Mood/Anxiety/Situational Stress (ρ = 0.45, *p* < 0.001) and those with psychosis (ρ = 0.39, *p* < 0.001).

**Figure 1:**
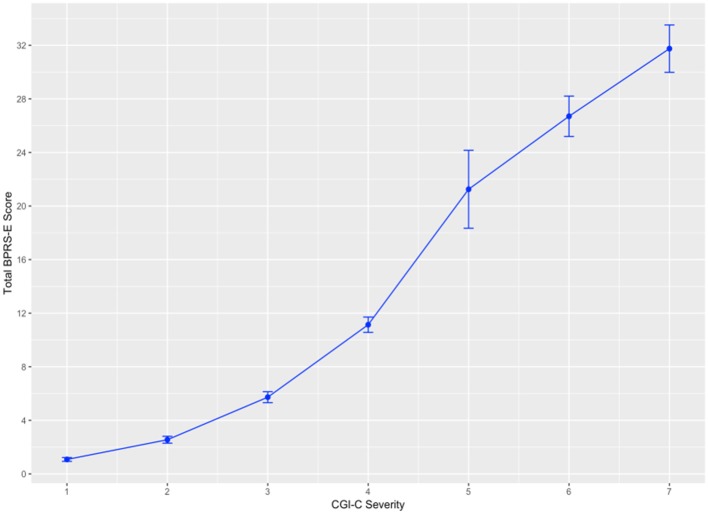
Linking between CGI-C and modified BPRS-E total scores for all patients. Error bars = SE.

Equipercentile linking between the CGI-C and the modified BPRS-E for the total sample are shown in Figure 1. Being scored as “Mildly ill”; a score of 3 on the CGI-C corresponds to the modified BPRS-E total score of 6. Scores of 4, 5, 6, and 7 on the CGI-C corresponded to total scores of 11, 21, 27, and 32 on the modified BPRS-E, respectively.

**Figure 2:**
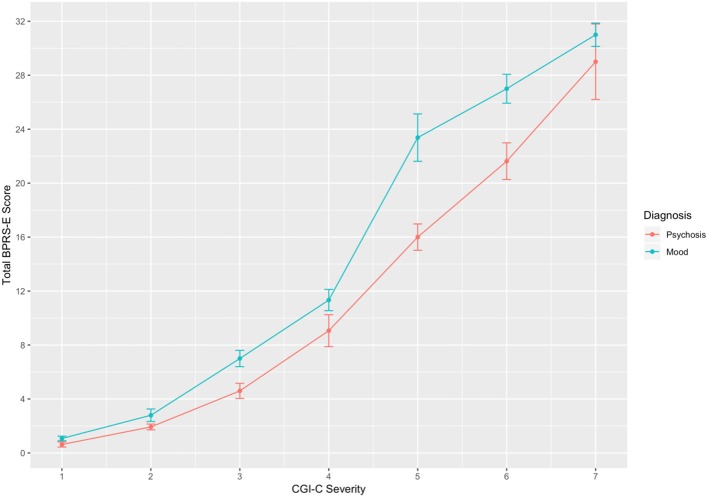
Linking between CGI-C and modified BPRS-E total scores for patients with a diagnosis of mood (*n* = 290) and psychosis (*n* = 185). Error bars = SE.

Figure 2 shows linking function performed separately for patients with a mood/anxiety diagnoses and patients with diagnosis of a psychotic disorder. A score 3 on the CGI-C corresponded to a total score on the BPRS-E of 5 for those with a psychosis diagnosis and 7 for those with mood/anxiety/situational stress diagnoses related disorder. CGI-C scores of 4, 5, and 6 corresponded to BPRS-E total scores of 9, 16, and 22, respectively, for patients diagnosed with a psychotic disorder and total scores of 11, 23, and 27, respectively, for patients with a mood/anxiety -related diagnosis. A total score of 7 on the CGI-C corresponded to a total modified BPRS-E score of 29 for those with psychotic disorders and 31 for patients with mood related diagnoses (see also Table 2).

**Table 2:** Equipercentile linked scores and standard errors between CGI-C ratings and BPRS-E total scale and putative diagnostic groups.

	**Linked BPRS-E Total Score (SE)**		
**CGI-C Scale**	**Whole sample (*****n*** **=** **726)**	**Psychosis (*****n*** **=** **185)**	**Mood/Anxiety/ Adjustment (*****n*** **=** **290)**
1: Normal, not at all ill	1 (0.99)	1 (0.19)	1 (0.17)
2: Borderline mentally ill	3 (0.22)	2 (0.21)	3 (0.46)
3: Mildly Ill	6 (0.36)	5 (0.56)	7 (0.60)
4: Moderately Ill	11 (0.76)	9 (1.18)	11 (0.79)
5: Markedly Ill	21 (1.43)	16 (0.97)	23 (1.76)
6: Severely Ill	27 (1.06)	22 (1.36)	27 (1.07)
7: Among the most extremely ill patients	31 (0.99)	29 (2.80)	31 (0.87)

## Discussion

We found that the CGI-C scores and modified BPRS-E scores among people in remand settings are significantly correlated and that points of equivalence can be reliably found between the two scales. This is the first study that has provided a validation of the CGI-C by demonstrating that clinician's global ratings correspond substantially with the total score on the BPRS-E, and the correlations between the two measures are identical for both men and women.

Although no study has previously compared the CGI-C with other symptom measures, comparisons of BPRS and CGI-S scores have been reported among patients with schizophrenia participating in drug trials (12, 21). We found that the Spearman correlation between CGI-C and modified BPRS-E (0.51) was similar to the mean baseline correlation between the BPRS and CGI-S reported in 14 drug trials among 4,065 people with schizophrenia (0.53) (12). Although in these studies there was concordance between CGI-S and BPRS scores, the authors cautioned that the results only applied to patients who have acute exacerbations of schizophrenia who have predominantly positive symptoms, and that the relationship between the two scales might be very different among those with primarily negative symptoms or other diagnoses.

No previous studies have reported equipercentile linkage of CGI-S and BPRS-E scores, however previous equipercentile linking of BPRS and CGI-S has shown a similar pattern to the findings in our study (19). Our study provides evidence that CGI-C has similar psychometric properties to the CGI-S among persons in correctional facilities, and with a variety of diagnostic groups. Our study also provides points of equivalence between the CGI-C and modified BPRS-E thus allowing for interpretation of studies that have reported results on one or other of the scales, and allowing for the intuitive application and interpretation of either scale among clinicians familiar with the other.

We found that the correlations between CGI-C and Mood/Anxiety/Situational Stress to be slightly higher (0.45) than among those with psychosis (0.39). We also found that the equipercentile linkage scores were somewhat different among the two groups. Among those with Mood/Anxiety/Situation Stress, scores on the CGI-C equated to higher BPRS-E scores than those with Psychosis, particularly among those rated as CGI-C 5, 6, or 7. Being considered moderately ill on the CGI-C equated with a BPRS-E score of 16 for those with psychosis and 23 for those with a mood related diagnosis, indicating that clinicians rate overall symptom severity higher among those with psychosis compared with mood and anxiety disorders with similar BPRS-E scores. This may reflect the more explicit focus on affective symptoms on the BRPS-E. The CGI-C may however better reflect the level of impairment caused by symptoms than the total BPRS-E scores as it relies on clinical judgement, and it has previously been stated that CGI is more informative than the BPRS as the ratings can be understood intuitively by clinicians (21).

With regards to limitations of our study, all inmates in our study were rated with both the BPRS-E and CGI-C by the same clinician. It would have been preferable to have had two clinicians independently rating either the BPRS-E or the CGI-C, and comparing those ratings, as it is possible that rating the BPRS-E may have influenced the CGI-C rating and vice versa when carried out by the same clinician, thus increasing the apparent correlation between the two. Our approach, however, would have reduced inter-rater measurement error that could be introduced from having multiple observers rating each person. It is notable however that the correlations we observed were comparable to those reported in similar studies. In addition, we have not measured the reliability of the putative diagnostic groups and therefore the differences in equipercentile ratings by diagnosis need further investigation for confirmation. We were not able to analyze patients by DSM-V diagnosis, but by symptom cluster as recorded in the JSAT. Future research could confirm our findings by investigating equipercentile linkage in more precise diagnostic categories. In addition, we were not able to analyze separately all of the symptom groupings recorded in the JSAT, such as intellectual disability or personality traits, as equipercentile linkage requires sufficient participants with recorded symptom severity across the whole range of possible scores in order to analyze.

Overall we found that the CGI-C ratings correspond very well to BPRS-E ratings for both men and women remand prisoners across diagnoses, and the CGI-C appears to be a valid tool for the assessment of severity of symptoms in this setting. It appears that the CGI-C is appropriate to be used as a routine measure of severity among both males and females. Further studies are recommended to assess the validity in other correctional settings to ensure generalisability, and to assess serial measures of the CGI-C to investigate sensitivity to change.

## Data Availability Statement

The data that support the findings of this study are available from the corresponding author upon reasonable request.

## Ethics Statement

Research Ethical approval was granted by the Centre for Addictions and Mental Health Research Ethics Board (#018/2018) for use of retrospective data for the purpose of research. No identifiable information was retained or is presented in this manuscript.

## Author's Note

RJ is a Forensic Psychiatrist and Clinician Scientist at the Centre for Addiction and Mental Health, Toronto, and is an Assistant Professor at the University of Toronto. CG is a Clinical Psychologist and Clinician Scientist at the Centre for Addiction and Mental Health (CAMH) and Assistant Professor at the University of Toronto. MM is a research coordinator at the Centre for Addiction and Mental Health (CAMH). AS is the Chief of Forensic Psychiatry and a Clinician Scientist at Centre for Addiction and Mental Health, Toronto, and is an Associate Professor at the University of Toronto.

## Author Contributions

RJ designed the study, carried out data collection, and preparation of the first and revised drafts of the manuscript. CG and MM contributed to the study design, carried out data analysis and interpretation of the results, and contributed to the preparation and editing of the manuscript. AS contributed to the interpretation of the results and editing of the manuscript. All authors have read and have approved the manuscript.

### Conflict of Interest

The authors declare that the research was conducted in the absence of any commercial or financial relationships that could be construed as a potential conflict of interest.
